# Imaging Diagnosis of Transient Ischemic Attack in Clinic and Traditional Chinese Medicine

**DOI:** 10.1155/2019/5094842

**Published:** 2019-02-17

**Authors:** Qigu Yao, Lincheng Zhang, Jing Zhou, Min Li, Weifeng Jing, Xiaohong Li, Jin Han, Lan He, Yuyan Zhang

**Affiliations:** ^1^Second Clinical Medical College of Zhejiang Chinese Medical University, Hangzhou 310053, China; ^2^College of Life Sciences of Zhejiang Chinese Medical University, Hangzhou 310053, China; ^3^College of Pharmaceutical Science of Zhejiang Chinese Medical University, Hangzhou 310053, China; ^4^Basic Medical College of Zhejiang Chinese Medical University, Hangzhou 310053, China

## Abstract

Neuroimaging plays a pivotal role in Transient Ischemic Attack (TIA). Generally, clinicians focus on the specific changes in morphology and function, but the diagnosis of TIA often depends on imaging evidence. Whereas Traditional Chinese Medicine (TCM) is concerned with the performance of clinical symptoms, they began to use imaging methods to diagnose TIA. CT and MRI are the recommended modality to diagnose TIA and image ischemic lesions. In addition, Transcranial Doppler sonography (TCD) and Digital Subtraction Angiography (DSA) are two acceptable alternatives for diagnosing TIA patients. This article elaborates the update of imaging modalities in clinic and the development of imaging modalities in TCM. Besides, multiple joint imaging technologies also will be evaluated whether enhanced diagnostic yields availably.

## 1. Introduction

Currently, cerebrovascular disease is the third commonest cause of death following malignant tumors and cancer, especially ischemic cerebrovascular disease, which has a high risk of paralysis [1].There are nearly 7.5 million Transient Ischemic Attacks (TIAs) worldwide each year [2]. TIA carries a particularly high short-term risk of stroke, and approximately 15% of diagnosed strokes are preceded by TIAs. Due to the negligence of TIA management, TIAs eventually evolved to stroke, which has brought huge economic losses and left the patients with disability and dependence [3, 4].

Clinicians tend to diagnose TIA with the collection of imaging evidence and duration of cerebral ischemia. With the increased recognition of TIA, update diverse imaging techniques have been used to improve the early diagnosis rate and accurate location of TIA, whereas TCM generally attributes TIA to the aura of stroke, starting from the earliest “The yellow Emperor' Classic of Internal Medicine” to the “Su Wen Men Tune Classics” and “Medical Forest Reform Mistakes” [5]. Nowadays, an increasing number of TCM doctors have found that imaging is more conducive to the classification and early identification of diseases, which is of great benefit to TCM treatment of TIA. In a word, both clinicians and TCM provide an overview that early detection and treatment of TIA are critical for potential stroke [6].

The largest obstacle clinicians and TCM doctors must overcome is how to confirm the evaluation and management of TIA with multiple neuroimaging technologies and joint application [7]. For example, cranial Doppler ultrasonography can be used for acute attack, as a minimally invasive method to identify large vessel occlusion or monitor stroke response. Compared with Digital Subtraction Angiography, four-dimensional CTA and MRA provide a less invasive alternative to determine the degree of vascular obstruction and collateral blood flow during macrovascular obstruction [8]. When there is substantial disagreement regarding TIA diagnosis, patients may miss the best treatment window and even get unnecessary treatment.

By combining different kinds of imaging, one may discover that neuroimaging focus cannot be separated from the three aspects of the ischemic penumbra (IP), cerebrovascular conditions (collateral circulation) (CVC), and cerebrovascular reserve (CVR). Further, if clinicians can obtain accurate imaging diagnosis of TIA, the prediction of early stroke and individualized therapeutic planning could be effective by implementation [9].

## 2. The Concept of Ischemic Penumbra, Cerebrovascular Conditions (Collateral Circulation), and Cerebrovascular Reserve

As early as 1977, Astrup defined the IP as perfused brain tissue at a level within the thresholds of functional impairment and morphological integrity [10]. It has the capacity to recover if perfusion is improved. It exists, even for a short period of time in the core of ischemia, from which irreversible necrosis propagates to the neighboring tissues over time. The IP between the ischemic core region and the normal blood flow region always exhibits dysfunction and electrophysiological disorders [11]. The main goal of TIA management is to prevent at-risk tissue from infarction by restoring blood flow to IP areas [12, 13]. It is now widely accepted that the IP is a necessary condition for thrombolytic therapy and is more effective to determine TIA therapy window [14].

Cerebral vascular conditions include the location of stenosis, the degree of stenosis, the time of formation, the collateral circulation, the luminal diameter, and blood flow. Some literatures point out that the establishment of good collateral circulation can minify low perfusion area and enhance the survival time of IP. Besides, it also contributes to endovascular treatment and reduction of infarct volume, which reduces the risk of recurrence and improve prognosis [15]. For clinics, the establishment of collateral circulation is also related to subsequent treatment [16]. The imaging results of collateral circulation determine the choice of interventional therapy or thrombolysis.

**Figure 1:**
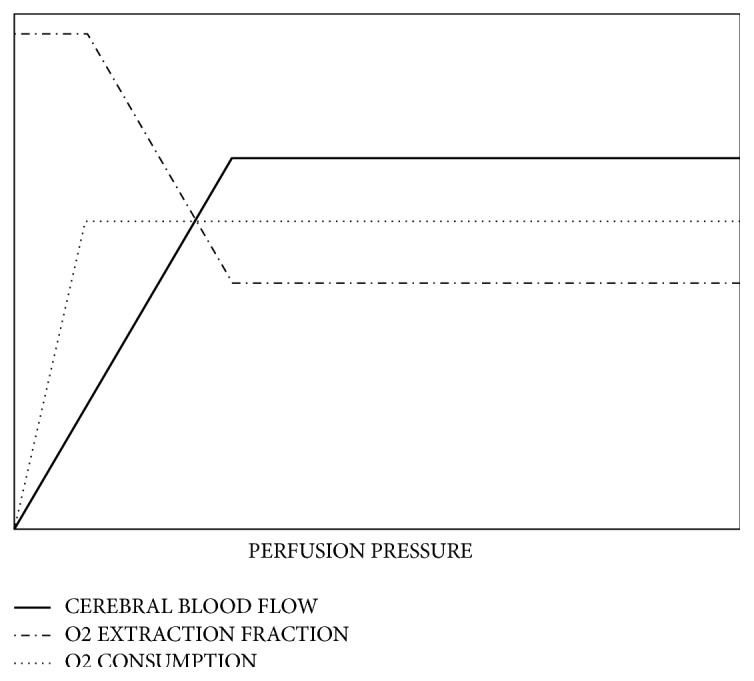
Given that idealized cerebral autoregulation. CBF, oxygen extraction fraction, and oxygen consumption as a function of perfusion pressure [10].

CVR refers to the intracranial arterial blood pressure changes, which can have semiautomatic self-regulation within a certain range [17]. The ability to maintain relatively normal cerebral blood flow can be used to evaluate the patient's risk status and it can be described as a function of perfusion pressures as shown in Figure 1 [18]. A study performed a total of 1061 independent CVR tests on 991 patients with an average follow-up of 32.7 months and found a significant positive correlation between CVR injury and stroke and the total randomized odds ratio was 3.86 (95% CI, 1.99-7.48) [19]. Current imaging evaluation methods include Positron emission tomography (PET), Xenon-CT (Xe-CT), and single positron emission computer tomography (SPECT). These techniques are used to evaluate patients by measuring the change of cerebral perfusion loading. It was found that CVR was an independent risk factor for ischemic stroke. And the measuring of CVR can predict the occurrence of stroke before clinical symptoms. For example, rapid occlusion of internal carotid artery (ICA) trunk may result in a significant decrease in hemodynamic reserve capacity before significant nerve injury occurs.

## 3. Multiple Imaging Technologies for TIA

### 3.1. Positron Emission Tomography

Positron emission tomography (PET) is a unique nuclear medicine test using positron emitters to show the pathophysiological process of the IP. Because of its long time and low resolution, PET is not suitable for the use of patients with acute cerebral infarction.

In the study of Sun, ischemic vascular diseases, including TIA and stroke, have been found that they are associated with elevated expression of *α*v*β*3-integrin via Positron emission tomography (PET). Therefore, PET provides a promising target for semiquantitative monitoring of the disease [21].

Several PET detection techniques include 15O-PET and 18F-fluorodeoxyglucose-PET. The former can quantify regional cerebral blood flow (rCBF), local cerebral blood volume (rCBV), local cerebral oxygen metabolism rate (rCMRO2), and local oxygen uptake fraction (rOEF). The characteristics of the central infarct zone are that rCBF, rCMRO2, and rOEF are declining. In the IP, rCBF generally declined, while rCMRO2 and rOEF increased, recognized as the gold indicator for determining the IP [22]. The latter focuses on rCMRO2, rOEF, regional cerebral metabolic rate for glucose (rCMRglc), and regional glucose extract fraction (rGEF). The mainstream of PET thinks these descendant indicators mean the location of IP.

Some experts emphasize that PET is the gold standard for cerebral blood flow reserve, because it can detect and quantify metabolic processes in cerebrovascular disease. Besides, PET can also reflect the change of blood flow, central nervous receptors, and the structure and function of brain tissue [23]. It is welcomed by patients and clinicians for PET's advantages, including its early safety, accuracy, rapidity, and noninvasive detection.

### 3.2. Single-Photon Emission Computed Tomography

The principle of SPECT and PET is similar, in which ischemic region is with low signal. Blood perfusion SPECT studies have shown that the upper and lower limits of rCBF in the IP are the 40% to 80% of the contralateral normal tissue [24]. Although the clinical application of SPECT is higher than that of PET, its shortcomings remain significant. SPECT has excessive time cost and does not provide cerebral blood vessel morphology, let alone radioactivity. SPECT is less sensitive than MRI for bilateral basal ganglia, because it uses the comparison of color grading by contrast agent. Cerebral blood flow imaging agents mainly accumulate in brain gray matter and thalamus nerve clusters, while white matter is so small that the brainstem and the bilateral ventricle become dead ends, thus SPECT has low sensibility [25]. According to a retrospective study of 107 patients with TIA, some scholars found that the positive rate of combined SPECT/CT diagnosis is 95.33% (102/107), which is higher than that of other single groups. CT technology may compensate for the shortcomings of SPECT images and detect ischemia and infarction early [26].

SPECT not only has a productive detection effect on IP but also a good reflection of CVR. Using the small molecules, such as 99Tcm-ECD, to pass through the blood-brain barrier (BBB) to stably stay in the ischemic tissue, SPECT can have early detection of ischemic lesions. Acetazolamide 99m Tc-HMPAO is used to form a parameter rCVR image and reliably evaluate hemodynamics of the internal carotid artery [27]. It has also been reported that SPECT imaging shows a fantastic predictive value for early reperfusion (within 2 hours) in a stroke experiment. In particular, there is a good predictive value of blood-brain barrier function, as well as the pathological changes of lung and intestinal inflammation [28]. Recently, researchers have proposed the combination of SPECT and computer aided analysis (CAA) methods and used the rate of change (CRM) image as a parameter image to quantify the change of local cerebral blood flow (rCBF) in longitudinal SPECT brain images [29]. The accuracy rate of CAA-CRM is 93.4% in 50 patients with TIA, and the clinical diagnostic value is high.

### 3.3. Computed Tomography

In recent years, with the continuous improvement of CT technology, a variety of CT technologies are widely used in TIA diagnosis. According to the patient's tolerance of contrast agents and risk assessment of prognosis, many techniques such as head CT, computed tomography perfusion (CTP), computed tomography angiography (CTA), or joint application are used extensively in order to obtain the comprehensive information of the patient at a time.

CT angiography (CTA) is a technique for displaying the shape of a blood vessel, such as a shape of a blood vessel and a stenosis by processing an image on a CT scan technique, when the contrast agent accumulates at the blood vessel defect [30, 31]. The use of CTA imaging in predicting stroke outcome has been investigated by several studies. Verro et al. reported that occlusion or high-grade stenosis on brain CTA in 56% of patients presents with acute stroke symptoms with a strong correlation with poor outcome [32]. In patients with acute stroke symptoms, clinicians are unable to differentiate stroke from TIA based on CT scan of the head. CTA has been used clinically to assess for significant stenosis/occlusion, and it has recently been shown to help prognosticate stroke and TIA patients. For instance, in a clinic study, the consistent rate of CTA and DSA results was found to be 88.9% [33]. However, another study demonstrated that CTA was assessed to have differences in the evaluation of collateral circulation. It is thought that the association with DSA is not high, even after combining CTA and CTP [34].

Using contrast agents, CTP performs dynamic scans to reflect intracerebral hemodynamics at selected levels [35]. The important parameters are cerebral blood flow (CBF), cerebral blood volume (CBV), mean transit time (MTT), time to peak (TTP), and peak enhancement (PE). With the advantages of plain CT scans, CTP is more accurate in the diagnosis of posterior circulation TIA, which can be confirmed in the follow-up examination of 3011 patients [36].The reduction of CBV and CBF often indicates the occurrence of cerebral infarction. When the CBF ratio of the affected side and the healthy side is less than 0.20, irreversible infarction occurs in the brain tissue. When the CBF ratio is between 0.20 and 0.35, some scholars believe that it is the range of the cerebral IP. MTT is a highly sensitive indicator of cerebral ischemia, but when determining early reperfusion, TTP and Tmax should be superior to MTT. Prolongation of TTP and MTT may be related to the establishment of collateral circulation [37]. Some studies suggested that CTA combined with CTP can improve the accuracy of predicting collaterals [34].

CTP imaging includes whole brain perfusion CT and dynamic perfusion CT [38]. The former provides cerebral blood volume (CBV), but it cannot measure CBF or MTT. The latter can measure CBF, CBV, and MTT but is limited to 2 to 4 brain slices currently [39]. CTP is part of the initial evaluation of stroke patients, allowing differentiation between the infarcted tissue and the IP. The clinical application of CTP is more and more extensive, and it takes less time and provides more information than MRI. However, a study found that when CT perfusion imaging (CTP) is used in the assessment of IP, it is often overestimated [40]. Qualitative evaluation of CBV and MTT maps may overestimate the real IP. MTT overestimated final infarct areas, because it may not differentiate true “at risk” penumbra from benign oligemia. Meanwhile, CBV overestimated the ischemic “core”, possibly due to delay in contrast arrival to the brain [41].

For CVR, CTP is used to detect. After cerebral ischemia, the corresponding level of CVR is developed and the cerebral vasculature compensatory is improved by increasing oxygen and glucose for neuronal cells in the pathophysiological process. The examination of CTP is very rapid, and the parameter MTT is highly sensitive to cerebral blood flow reperfusion. And the degree of impairment of CVR is closely related to the change of MTT, which was observed in a study from August 2009 to August 2014 [42]. Wang summarized that changed parameters that MTT prolonged, CBV increased, and oxygen uptake fraction (OEF) invariant indicates vascular reflex expansion; CBF decreases, OEF increases, and means brain metabolism reserve mechanism begins to take effect [42]. One study used 48 mice to compare CTP, Triphenyltetrazolium chloride (TTC) staining and hematoxylin-eosin staining, and CTP showed consistent infarct volume calculated in TTC staining and CBV showed the highest correlation [43]. Since CTP has many advantages, it has also substantial shortcomings such as low specificity of the examination, large differences, and limitations in the perfusion parameters [44]. In future, subsequent prognostic studies should be done and the difficulty is how to obtain more layers of thick brain tissue.

### 3.4. Magnetic Resonance Imaging

Magnetic resonance imaging (MRI) is also commonly used to detect IP [45, 46], CVC, and CVR. Besides, MRI has multiple patterns to obtain optimal assessment for patients who are stratified by the individual risk profile. The advantage lies in its wide applicability, but studies have suggested that it has an overestimation of the IP or changes in CBF. Furthermore, the patient's movement may produce a false impression and the prohibition of metal in the patient is also limited.

Perfusion-weighted Imaging (PWI) is a widely applicable clinical tool with high accuracy [47]. The imaging principle of PWI is that local mean transit time (rMTT) is equal to local cerebral blood volume (rCBV)/(rCBF), aiming to reflect changes in brain microvascular morphology and brain microhemodynamics [48].

When it comes to CVR, there is no significant difference between PWI and CTP. rMTT can reflect the measurable changes in local blood flow on ipsilateral and unaffected brain tissue and find early ischemic tissue. With regard to recurrent cerebral infarction after TIA, previous study found that 30% of acute focal PWI lesions after TIA are associated with new brain infarction (BI) at 1 week [49].

**Figure 2:**
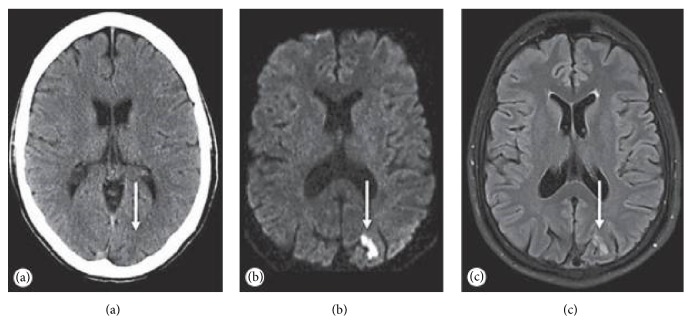
CT and MRI findings in a 49-year-old TIA patient. CT (a) diffusion-weighted (b) and T_2_-weighted FLAIR MRI (c). (b and c) Show a small acute ischemic lesion (arrow) in the left posterior cerebral artery territory. However, CT is not clearly [20].

Diffusion-weighted Imaging (DWI) can accurately follow the diffusion water molecules and show acute ischemic changes within a few minutes after vascular occlusion [50]. The apparent diffusion coefficient (ADC) derived from the DWI sequence and this biomarker has been proved to discriminate between normal, ischemia, and infarcted regions in stroke. Lopez-Mejia analyzed three hundred ADC measurements, 100 from each selected region (infarct, penumbra, and normal brain regions). There was a statistically significant difference in ADC values for the 3 regions. By comparing ADC measurements, radiologists might discriminate penumbra regions from infarcted brain tissue, which might provide additional information to tailor follow-ups and treatments in specific populations [51]. Furthermore, DWI has a better detection speed than CT, according to a comparison between computed tomography and magnetic resonance imaging made by Froster [20]. DWI confirmed the acute infarct on CT in only 2 of the 7 patients (28.6%), 1 in the anterior circulation, and 1 in the posterior circulation, respectively (Figure 2). The American Stroke Association Committee also recommends that, for patients with TIA symptoms, DWI should be considered as the first choice for its high sensitivity to detect acute brain injury. And it is confirmed in the CT/MRI retrospective cohort of 7889 TIA patients by Seemant Chaturvedi et al. [52].

The “mismatch” between perfusion weighting and diffusion-weighted abnormalities is an indicator of the IP. However, some scholars have found that PWI often overestimates the risk of stroke in patients. Because the tracer cannot be determined accurately in pathological perfusion tissue, the obtained absolute or relative thresholds of PWI/DWI cannot be completely reliable [53, 54].

Magnetic resonance spectroscopy (MRS) is also a type of MRI that can noninvasively and dynamically measure the levels of major metabolites in the brain [55, 56]. It mainly detects levels of N-acetyl aspartate (NAA) and the degree of lactate complexes (Lac) to reflect brain tissue activity. The different local oxygen content in the infarct core, penumbra and nonlesion area, and local lactate (Lac) is also different. Lac wave can be detected with 1H-MRS, and the Lac peak in the core infarct zone is high. And the Lac peak of the surrounding area in core infarct zone declines, and that area is what we call IP. In the early stages of infarction, the Lac peak appeared earlier than NAA, and its peak in the cortex is higher than subcutaneous tissue, particularly in the cortex or the internal and external capsule regions. Therefore, the level of Lac/NAA can be used to distinguish PWI/DWI mismatch regions from suspicious infarcts [57].

Susceptibility weighted imaging (SWI) displays phase maps through the characteristic magnetic susceptibility differences of adjacent tissues, which is extremely sensitive to venous blood vessels, blood products and vascular malformations [58, 59]. Unlike MRA showing arteries, SWI mainly uses the reflection of veins to determine the degree of brain tissue oxygen tolerance [60]. The oxygen extraction fraction (OEF) has a low signal vascular sign at the ischemic end, analyzed by Vural et al. [61]. And they found that the cortical vein signal was lower in areas with better collateral circulation.

### 3.5. TCD: Noninvasive Diagnosis

**Figure 3:**
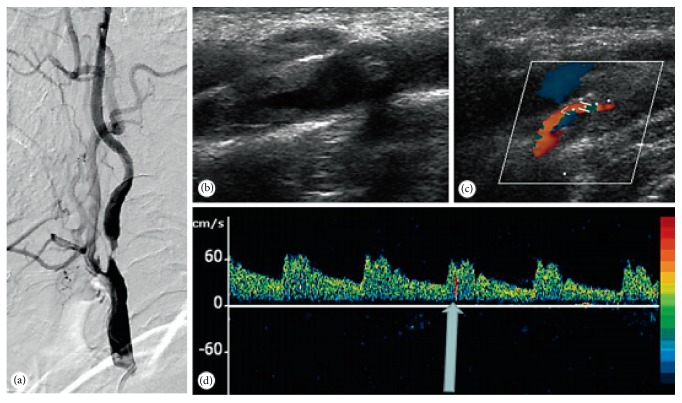
A 67-year-old male with recurrent TIAs. However, DSA (a) and TCD (b and c) demonstrated a severe focal stenosis of the proximal ICA. Despite initiating aspirin, patient TCD monitoring of the left MCA demonstrated spontaneous MES (d).

Transcranial Doppler sonography (TCD) is used to detect intracranial and extracranial arteries hemodynamics, blood flow direction, and spectrum morphology [62]. Advanced applications of emboli monitoring, vasomotor reactivity, and detection of right-to-left shunts (RLS) help in understating the enteropathogenesis of cerebrovascular ischemia (Figure 3). It is sensitive to accurate detection of cerebral vascular stenosis, sclerosis, spasm, and occlusion, which is widely used in clinic [63, 64]. TCD can reduce the patients at the risk of radiation exposure, which is price-friendly, noninvasive, and simple [63]. A recent clinical trial demonstrated that TCD was very useful for the posttreatment of patients with recurrent syncope, and it was a noninvasive technique that provides real-time measurements of cerebral blood flow velocity [29, 65].

For TCD examination of the first-stage collateral circulation (Circle of Willis), the blood flow is often reversed on the lesion side [66]. Meanwhile the blood flow velocity on the contralateral side is often increased. For example, ischemia of the internal carotid artery, the ophthalmic artery, and the anterior and posterior communicating arteries will rapidly open to cover the ischemic area [67]. Besides, some studies have found that TCD assessment is useful in patients with anterior circulation than whose with posterior circulation, but it is still difficult for clinicians to find the regional ischemia on the lesion side and the increased flow velocity on the contralateral side without well-trained operating technique [68]. The assessment of secondary collaterals by TCD refers to the compensatory reflux and blood flow of the ophthalmic artery, when the internal carotid artery is narrowed. TCD showed difficulty in the pial lateral and tertiary collateral vessels; especially intracranial vascular stenosis <50% was less easily detected. The accuracy of collateral circulation was lower in elderly and female patients. Moreover, it cannot show the morphology of cerebrovascular vessels [69, 70].

### 3.6. Digital Subtraction Angiography: A Gold Indicator for TIA

Currently Digital Subtraction Angiography (DSA) is recognized as a gold indicator for detection of vascular stenosis, flow velocity, blood flow, and establishment of collateral circulation [71]. Common examination sites for TIA include ACA (anterior cerebral artery), MCA (middle cerebral artery), PCA (posterior cerebral artery), OA (ophthalmic artery), and BA (basal artery). With the wonderful intuitiveness of blood vessel and high accuracy, DSA is considered to have the highest diagnostic value in TIA patients. However, the problems with DSA cannot be ignored [72]. For instance, firstly, DSA is an invasive method of examination with excellent sensitivity, but its risk implies it should not be used as a preferred item for patients [73]. Secondly, the cost of DSA inspections is still a factor that cannot be ignored [74]. Thirdly, the difficulty of DSA operation, including requirements for contrast dose and pressure for injection, is also a major challenge for clinicians [75].

## 4. The Use of Radiology in TCM

Given the diagnosis and treatment of TIA with TCM is often judged by its clinical symptoms and age first, typical symptoms include partial paralysis, numbness, dizziness, and acute exacerbation of speech or repeated attacks. From the respective of TCM, the tongue coating is more common with thick or greasy tongue in TIA patients [5]. In the past, TCM doctors only relied on clinical symptoms to diagnose TIA patients and judge prognosis. It is not very accurate and often depends on the accumulation of experience. The development of imaging is a useful and accurate supplement for TCM doctors to diagnosis of TIA. The imaging in TCM focuses on the determination of classification, the evaluation of clinical therapeutic effect of TCM and the combination therapy of TCM and Western Medicine.

The common imaging techniques used for the discrimination of symptoms are mostly MRI and CT. The study of the relationship between the syndrome types and the change of cranial blood vessels in TCM will help have accurate dialectical treatment of TCM and improve the cure rate for individuals. In a clinic study, clinicians used CTA to find that there were more plaque formations in the wind phlegm obstruction with qi deficiency and blood stasis syndrome [76]. It has also been found with the development of single or composite clinical model, such as blood stasis and wind palpitation, that imaging has its own characteristics of high reliability [77]. Combined CTA and CTP technology, researchers found that the proportion of qi deficiency was highest in patients with TIA based on wind, sputum, qi deficiency, and blood stasis and other six kinds of syndromes, and the proportion of qi deficiency is 58.3%, respectively [78]. Imaging techniques are used to clarify the adhesion of platelets, the accumulation of protein, and intracellular acid-base, which improve the accurate classification of TIA patients.

In addition to the use of imaging methods to assist in the analysis of patient classification, TCM doctors have used imaging methods to judge the therapeutic effects of TCM, which has been widely used in the treatment of Chinese medicine. The specific indications, contraindications, and characteristics are similar to the above, so they will not be repeated.

In one study, researchers compared the TCM and clinical treatment of 128 TIA patients who depend on imaging techniques and TCM syndromes to attain diagnosis and classification. They found that the effective rate in the treatment group with clinic basic therapy and TCM was 96.9%, which was much higher than 87.5% in the control group with only clinic basic therapy [79]. The combination of traditional Chinese and Western medicine is a promising way to treat TIA patients. And the accuracy of diagnosis and classification is helpful for TCM doctors in using different prescriptions for TIA patients in order to enhance effect.

The auxiliary imaging diagnosis of Chinese medicine dialectical treatment is particularly reliable in TCM. Although the combination of TCM and radiology is still at a relatively early stage, the remarkable results of Integrated TCM and Western medicine treatment have shown great potential, which have been demonstrated in relevant literature [80]. According to the recommendations of the guidelines for the diagnosis and treatment of cerebral infarction with Integrated TCM and Western medicine, the discrimination of diseases in clinic and the discrimination of symptom in TCM is most worthy of attention [81]. We now have a bold prediction that various imaging techniques will be used in TCM dialectic and the combination treatment of TCM and Western medicine in the future.

## 5. Conclusions

At present, there are more and more methods for the diagnosis of TIA in TCM and clinic, and imaging has always occupied an extremely important position. Even with the constant development of software and hardware, it has become too increasingly important to ignore. The three-dimensional, clear, dynamic, and automated images will be the future trend. The new TIA metabolites and iconic marker will be the breakthrough, aiming at individualize treatment of TCM and clinic. Based on the recent research hotspots, three aspects of the IP, CVC, and CVR are still hot areas for future research.

**Figure 4:**
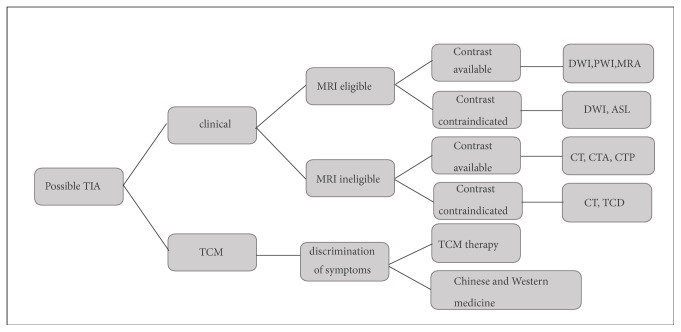
TIA imaging strategy. For the diagnosis of TIA, clinical diagnosis is still the mainstream diagnosis approach, while TCM diagnosis is a useful supplement. The application of various imaging techniques needs specific analysis because of its unique advantages.

Computed tomography (CT) is widely used to detect early signs of ischemia. CTA is helpful for physicians to find evidence of intravascular thrombosis or significant stenosis with patients who suffer acute ischemic. Meanwhile CTP usually shows various parameters of ischemic brain tissue and evaluates the salvage value. MRI is more sensitive than CT in diagnosing acute cerebral ischemia, especially in the DWI hyper acute period. As a noninvasive and nonionizing imaging method, MRA is used as an alternative to CTA and received the admiration of the patients and emergency department physicians. The sequences of TIA patient diagnosis approach we recommend are a combination of the Western Medicine and Traditional Chinese Medicine and multiple imaging based on individuals (Figure 4) [82]. In the future, by combining different imaging techniques in a multimodal approach, we can obtain the needed information for treatment planning and distinguish patients who require thrombolytic therapy [6].

In conclusion, the complexity of the patient's condition determines that multiple imaging diagnostic modality should be used in TIA patients. The combined use of multiple imaging methods will be able to solve the current “individualized” needs of patients for treatment and provide sufficient basic work for targeted and advanced treatment plans [83]. We confirm that reasonable imaging studies in clinical work will provide an objective and valid basis for TIA diagnosis of TCM and Western medicine.
